# Male involvement in maternal healthcare through Community- based Health Planning and Services: the views of the men in rural Ghana

**DOI:** 10.1186/s12889-017-4680-2

**Published:** 2017-09-06

**Authors:** Bassoumah Bougangue, How Kee Ling

**Affiliations:** Faculty of Social Sciences, Universiti Malaysia Sarawak, Kuching, Sarawak Malaysia

**Keywords:** Male involvement, Community-based Health Planning and Services, Traditional gender norms, Women’s roles, Perpetuation and reinforcement, Maternal healthcare

## Abstract

**Background:**

The need to promote maternal health in Ghana has committed the government to extend maternal healthcare services to the door steps of rural families through the community-based Health Planning and Services. Based on the concerns raised in previous studies that male spouses were indifferent towards maternal healthcare, this study sought the views of men on their involvement in maternal healthcare in their respective communities and at the household levels in the various Community-based Health Planning and Services zones in Awutu-Senya West District in the Central Region of Ghana.

**Methods:**

A qualitative method was employed. Focus groups and individual interviews were conducted with married men, community health officers, community health volunteers and community leaders. The participants were selected using purposive, quota and snowball sampling techniques. The study used thematic analysis for analysing the data.

**Results:**

The study shows varying involvement of men, some were directly involved in feminine gender roles; others used their female relatives and co-wives to perform the women's roles that did not have space for them. They were not necessarily indifferent towards maternal healthcare, rather, they were involved in the spaces provided by the traditional gender division of labour. Amongst other things, the perpetuation and reinforcement of traditional gender norms around pregnancy and childbirth influenced the nature and level of male involvement.

**Conclusions:**

Sustenance of male involvement especially, husbands and CHVs is required at the household and community levels for positive maternal outcomes. Ghana Health Service, health professionals and policy makers should take traditional gender role expectations into consideration in the planning and implementation of maternal health promotion programmes.

## Background

The need to promote maternal health in Ghana led to the government’s commitment to extend maternal healthcare services to the door steps of rural families through community-based health planning and services (CHPS). The CHPS programme uses community health officers (CHOs) to provide a range of services including antenatal care (ANC), postnatal care (PNC) and emergency delivery [1, 2]. By reducing geographic barriers to healthcare, the CHPS strategy enables the Ghana Health Service (GHS) to reduce health inequalities and promote equity of health outcomes [2, 3]. The implementation of CHPS requires the cooperation of the health sector and involvement of all stakeholders, especially men, in the provision and reception of healthcare [3].

The need for male involvement in maternity care can be traced back to the 1994 International Conference on Population and Development [4] in Cairo, that emphasised the inclusion of men as partners and role players in maternal healthcare. In recognition of the importance of male involvement, the strategic plan of the Ghana Health Service emphasises the involvement of males, especially in the CHPS programmes aimed at promoting maternal health at the household and community levels. However, little attention has been given to male involvement in maternity care at the household level, nor have adequate studies been done to explore the extent and nature of male involvement in maternity care in the CHPS zones.

Even though men play a key and vital role in the Ghanaian family system, especially in patriarchal societies, as the main decision-makers and providers of resources and funds during pregnancy and childbirth, the existing maternal healthcare programmes tend to focus more on the role of pregnant women. In a previous study conducted by the lead author [5] in the Awutu-Senya District, some women raised the concern that their husbands were indifferent towards assisting in maternal healthcare. Yet, interaction by the lead author with their husbands, showed that they were concerned about the health of their wives and babies. Another study showed that men were not actively involved in maternal healthcare programs under the CHPS initiative [6]. The questions arising are therefore: to what extent are the women’s husbands involved; and what is the nature of their husbands’ involvement?

Male involvement has been defined variously, depending on the focus of the study [7–10]. In the Ghanaian setting, male involvement in maternal healthcare has been described as the involvement of men in clinical attendance with their spouses and the participation of men in traditionally female gendered roles such as cooking, washing, fetching of water at the household level [7–9]. Other research has considered men’s provision of funds for clinical care as indicative of male involvement, although free maternal health care has been introduced in recent times [7, 11, 12]. Evidently, there is no consensus on the definition of male involvement, although consensus could provide a framework for describing distinct domains of male involvement [11].

This paper is based on a study which explored the views and experiences of men in relation to their involvement in maternal and child health care programmes at the local community level. The main objective was to provide opportunity for men to share their views about involvement in various aspects of maternal health care, ranging across pregnancy, delivery and postnatal care.

This study has made a small yet significant contribution in bringing out the hitherto unheard views and experiences of men in relation to maternal healthcare involvement. It was able to obtain insightful data which pointed towards socio-cultural and institutional factors that influence the nature of male involvement. This in turn has far-reaching implications for planning and implementation of maternal health promotion programmes.

## Research design

The study adopted a qualitative research method, as the primary aim of the study was to explore the views and experiences of men’s involvement in pregnancy-postpartum care. This design was adopted to provide a space for men to share their views through focus groups and individual interviews in order to capture the underlying issues. Thematic analysis was used to analyze the data which was categorized under socio-cultural, institutional/structural and socio-demographic domains.

## Methods

### Study setting

The Awutu-Senya West District is one of the newly established districts in the Central Region of Ghana with Awutu-Beraku as the administrative capital. This district, which has several sub-districts, used to be part of the then Awutu-Senya District. There are four public health centres located in Awutu-Bawjiase, Senya-Beraku, Awutu-Beraku, and Bontrase and five Community Based Health Planning and Services (CHPS) zones located in Papase, Akrabong, Tawiakwaa, Mayenda and Okwampa. There are 15 privately owned health institutions in the district. However, there is no district hospital. The health centres provide medical care, communicable disease control, family planning, reproductive services, nutrition and post-natal services [16]. The district depends on hospitals in neighbouring districts for referral and specialist services in Agona Swedru and Winneba.

### Sampling and data collection

The participants were selected from the various communities to which the five CHPS zones in the district are assigned. The main participants in the study were spouses of women who had been pregnant at any time between 2008 to 2010. The emphasis of the study was on the most recent pregnancies, based on the assumption that participants would be more able to recollect their experiences from more recent events. Other participants were the main stakeholders of the programme at the community level such as traditional leaders, community volunteers and community health officers (CHOs) who represent the Ghana Health Service and Ministry of Health. In all, 93 husbands were involved in the study. The study drew on ten focus group discussions (FGDs) involving 68 husbands and 25 individual interviews involving husbands from the selected communities. The use of both focus groups and individual interviews was to ensure validation of data by taking care of the limitations of either methods. The 25 husbands included 2 husbands from each of the five CHPS zones, 5 traditional leaders and 10 CHVs. Additionally, 5 CHOs, at least one from each CHPS zone, were interviewed individually. This involved 30 individual interviews altogether.

Ten focus group interviews with husbands were organised in the five CHPS zones, with each zone having two such group interviews. The focus groups were composed of men from various communities which the CHPS facilities serve, to ensure wide representation. Participants for the focus groups were segregated according to age, place of residence and level of education, to allow for homogeneity so that participants of common interest could discuss issues freely. This was also arranged with the view to comparing the involvement of men from remote areas with those from communities in which the CHPS facilities were sited.. The individual interviews also catered for men from various communities as well as from various types of relationships - formal marriages or cohabitational relationship, monogamous or polygamous marriages or relationships. Individual interviews were also conducted with various categories of people and stakeholders in the programme such as CHOs, volunteers and traditional leaders, on the strategies and activities they undertake to actively involve spouses in the maternal and child health care programmes.

The participants for the research were purposively selected. Clinical records of their spouses in various health facilities were used to make contact with the men. The lists of contacts of CHVs were provided by the CHOs and the traditional leaders of the communities. The snowballing technique of sampling was employed to reach the targeted participants, whilst the quota technique was used to select participants based on special background characteristics of interest such as age, residence and educational attainment. This ensured wider representation in the data and analysis. The interviews, which were conducted in the local Awutu dialect and in English Language, were recorded with a tape recorder and transcribed. The Awutu dialect was later translated into English Language by the first author, who is the main researcher of this study.

### Ethical consideration

Before data collection began, the researcher contacted the district health directorate and the community gates, explained the purpose and usefulness of the research to them and sought permission from them to collect data. The participants were also given consent forms to read and sign. Consent forms were read and explained to those who could not read and they were invited to use thumb printing, signifying their understanding and agreement to take part in the exercise. Also, the consent of the participants was sought for publication of the study outcome for public consumption. The interviews were organised in familiar environments, conducive to relaxed participation. This motivated participants to contribute actively as they observed the basic rules and principles of giving respect to the views of co-participants in the discussions. The researcher also assured participants of anonymity and confidentiality, which motivated them to talk more freely in the interviews.

### Data analysis

The study was programmed to allow for early expansion of field notes and transcription of recorded data in the first interviews of FGD and IDI to serve as a guide for subsequent data collection.

Thematic analysis was employed for analysing the data. Data which was recorded with a tape recorder during the FGDs and IDIs sessions was played and listened to repeatedly in order for the researcher to become familiar with the data. There was a comparison of words, emphasis of respondents, comments, consistency of comments, and the specificity of responses in follow-up probes. Similar thoughts expressed across the participants’ responses were identified, coded and grouped together. Out of each group of similar thoughts, a unifying concept or underlying theme was derived. Key points, phrases, and illustrations were also identified to back up the findings. Finally, emerging themes that were similar were grouped together to identify major themes, through a consultative process amongst the authors.

## Results and discussions

Generally, the data indicated that men were involved in some ways in maternity care both at the household and community levels. At the household level, men were involved in settling medical bills but admitted that they were not involved much in household chores throughout their wives’ pregnancies and postpartum periods. Some men admitted that women undergo a lot of suffering during pregnancy and childbirth; however, they explained that they could not do anything to help, since pregnancy, childbirth and household chores are women’s roles. Nonetheless, several husbands who had been helping their wives, expressed concern about wanting to assist their wives during the pregnancy-postpartum period. They argued that, even though pregnancy is not a sickness per se, it is normally associated with complications and for that reason men should help women during this period.

At the community level, these men have contributed to the building of health facilities, which they explained could not be undertaken by women; nor did they expect the government to be responsible for building these facilities for them. This finding corroborated with a study in the Volta Region of Ghana on male involvement, which showed that men were mostly involved in supporting their spouses financially to seek ANC but their direct involvement in delivery and post-delivery care was low [13]. This is similar to the findings of the annual health sector review in Ghana which established that men were not engaged in activities of CHPS programmes and were solely involved in communal labour in the CHPS zones [6].

The following are further discussions of the findings under three main topical areas, namely: socio-cultural (traditional gender role expectations, the need for harmony and marriage sustenance and worldviews and beliefs); institutional (community and organisational factors); and socio-demographic factors.

### Social and cultural factors

#### Traditional gender role expectations

The socio-cultural factors that have been identified are very much centred around traditional gender role expectations. Helping in household chores and accompanying spouses to clinics for check-ups or deliveries were welcomed opportunities for a few men. Most of the husbands expressed the view that there are roles for men and roles for women. Premised on their culture and religious beliefs, this issue generated hot debates amongst the participants in the FGDs. Whilst those advocating for helping in household chores claimed it would reduce women’s burden and make the women and their foetuses healthy for safe delivery, others claimed that for a man to be cooking when the wife is around is culturally unacceptable. They argued that, where a woman is unable to work during pregnancy, female relatives need to come and stay with her or the woman has to go and stay with her parents for the mother to help her. This is what a 49-year-old husband and driver, who is a graduate of Junior Secondary School, said:

“*I think our forefathers know that men and women are different. That’s why they make them wives go and stay with their mother there to give birth. So, pregnant woman must go and stay with her mother.*”

To some husbands, women who preferred to stay with them instead of with their mothers or any other female relatives during pregnancy might have been worried that their husbands would become interested in other women during the period. This issue was raised by most participants in FGDs, supported by many participants in each group. The same 49-years-old man added:

“*But some of the women say if they go away, their husbands will take another woman, so want to stay with their husbands and suffer and give problems. So, if they stay with them husband it means they strong and fit and can work.*”

Whilst some of the men claimed that after marriage a man and a woman become one and need to help each other, others held the view that a man cooking and washing tarnishes the image of the man in the eyes of the community. Further probes into the issues revealed that men who tried to help their wives in household chores were branded as “okotobonku” (a local name for a man always identified with women or found to be playing the role of women) or “tok-tok” (stupid).

In helping their wives, men received a lot of uncomplimentary comments from colleagues, and family members, especially their own mothers. If they persisted in performing such “women’s tasks” they would lose respect from friends, family and the community at large. A few men revealed that men who were sweeping, cooking and washing were not given a listening ear in community meetings and allowed to make decisions because other meeting participants considered their contributions were coming from their wives. In fact, some men expressed that they would want to offer help to their wives, especially during the period of pregnancy and childbirth, but the social and cultural structures in the communities were not conducive to them performing such tasks. In an IDI, a 50-year-old man, who is a middle school certificate holder, said:

“*As a man and the head of the family, you need to protect these norms and have the respect to be able to train your children. Because if the community members do not respect you, your children may also not respect you and you cannot control them.*”

Similarly, gender role expectations appeared to be strongly held by the women themselves. In the individual interviews, one participant aged 40 years, who is a carpenter and secondary school leaver, lamented:

“ *I thought I was helping my wife to keep her healthy when she was pregnant and weak not knowing I was losing the respect people have for me and degrading myself. I only realised this when I had a misunderstanding with a community member and she told me that I only attended secondary school to learn how to perform female roles. She called me man woman” (Christian)*.

Leaders in four of the five selected zones also noted that the behaviour of some women was a major deterrent to male involvement, especially in household chores. In all the study communities, there were names given to men who performed household chores or accompanied wives to clinics. According to the community leaders, some women did not respect men who performed these duties. This is what one community leader noted in IDI:

“*It is unfortunate that women themselves create their problems by defining roles for themselves and giving female names to men who help their wives at home. As a chief, I have settled a number of cases where women insulted men for performing the traditional roles of women. They give all sort of names to them” (middle school leaver; 53 years old; chief).*

Gender role expectations restricted men’s involvement in maternity care because the society had no space for men in such “feminine roles”. This concurs with previous observations in Ghana [7–9, 11, 20] that the traditional gender norms have been internalised and normalised in these communities. As discussed by several researchers in Ghana and other African societies [14–17], these cultural norms and values exerted an influence on men’s behaviour in the family sphere and in this case, in the way they are involved in maternity care. This suggests that, to succeed in maternal health care programmes, there needs to be calls for recognition on the part of the policy makers, implementing bodies and researchers of the influence of these cultural norms, before they are able to adopt ways of overcoming the obstacles towards change [13, 18, 19].

#### The need for harmony and marriage sustenance

The data from the study indicated that some husbands tried to avoid performing households chores and accompanying wives to health facilities in order to promote harmony and sustain their marriages. According to the men, there were cases of some women who were known to deceive their husbands by collecting money from them for purchasing items related to maternal care, check-ups or treatment but instead spent the money on different things not accounted for. So, if men tried to shop for their wives or follow them to the health facilities for check-ups, some women felt their husbands did not trust them. Both in the FGDs and IDIs, the husbands explained that, as a way of promoting harmony and ensuring sustenance of their marriages, they had to totally avoid performing these roles. It was reported that men who shopped for their wives or followed them to clinics to settle bills were given names such as “pepei” or “iron- handed man” (suggesting that they are stingy). It means they did not want their wives to spend their money. A participant who had a misunderstanding over the same issue with his wife, shared this in IDI:

“*If you advise the cat try as well to advise fish (a local proverb literally meaning that both men and women are to be blamed). Some women think helping them to the hospital is to cut down expenditure because that is the period they normally collect monies that cannot be accounted for from their husbands. Could you imagine that in an attempt to accompany my wife to hospital in her last pregnancy we fought? So under this condition how do you expect men to help women?*” *(37 years old; junior high school graduate; farmer).*

According to the participants, sometimes, not following their wives to the health facility or shopping for their wives during maternity, is a way of demonstrating their trust in them. However, some participants who disagreed with their colleagues said they did not care about what the society or their wives would think about them because whatever action taken within the period, in the form of help, was in the interest of their wives and the expected children or newborns. In expressing disagreement in FGD a participant said:

“*It is the life of the unborn child and that of the mother that matters and not what my wife or the society says.*” *(46-year-old man and farmer; no formal education).*

Women’s behaviour was identified as an important issue which informed husbands’ assistance in household chores. Regardless of the socio-economic status of the wife or her age, husbands’ contributions in household chores sometimes depended on the women’s behaviour towards them. Most of the participants expressed that ‘respectful, humble and submissive’ wives most often received assistance from their spouses, even when they were not pregnant or sick. The participants said that, after pregnancy and childbirth, some women still expected their spouses to perform women’s roles when they were fit or not pregnant. They claimed that, once they started undertaking women’s work, the women would expect it to continue and failure to comply would always result in misunderstandings between spouses. Therefore, to avoid this issue, some of the men tried not to involve themselves at all.

It is interesting to note that the views that women had to be ‘respectful, humble and submissive’ to deserve support, came from men who married women from matriarchal communities. The Awutus inherit matrilineally so some women own lands and are economically empowered. Still, a few other participants mentioned that their wives provided lands for farming, and housing. These lands were inherited properties from the women’s lineage. Husbands of women who were engaged in trading noted that they needed to help their wives, as the women also contributed to the payment of bills, including school fees for their children. It was observed that men looked for qualities such as humility, submissiveness and respect from their wives if they were to be actively involved in performing traditional roles of women. Thus, a woman may have been empowered economically and politically, but without these qualities the husband may not have helped in performing “women’s roles”. In an IDI a 29-year-old taxi driver said:

*“It is my wife who created this job for me by buying this taxi for me. Yes, I helped my wife whenever she was pregnant. It is not because she bought a taxi for me. It is my responsibility. The motivating factor is that irrespective of her wealth, she respects me as a husband. Helping women during pregnancy in household chores and accompanying them to clinics is a way of showing love and care and that is the beginning of responsible parenthood. However, only humble, submissive and respectful women deserve that (senior high school graduate).”*

#### Beliefs related to infidelity and prolonged labour

The perceptions and beliefs that the men had about male involvement in maternal healthcare influenced them in their involvement. Most of the participants believed that any woman who had engaged in extra marital affairs would experience prolonged labour and until she confessed, she would not have a safe delivery. Therefore, following wives to hospital was a particularly serious taboo in patriarchal societies and children from extra marital pregnancies were not regarded as true blood of the family. Therefore, in patriarchal societies, men assigned their mothers or sisters to accompanying their wives to clinics. Based on this, relatives of the husband had to witness delivery to be sure that the child was their blood, in the event of any confession.

However, men in a matriarchal society assigned their mothers-in-law and sisters-in-law to taking care of their wives during pregnancy and delivery. Irrespective of which man was responsible for the pregnancy, children in a matriarchal society followed the mother’s family lineage. Once the child was born, he/she was recognised a member of the mother’s family where he/she could inherit property. Even though extra marital relationships were not permitted in either patriarchal or matriarchal societies, children born in patriarchal societies particularly suffered from a lack of care from fathers and were not recognised as belonging to either side of the family. In the IDI a 53-year-old man said:

*“In this community, if you go to the school you will find that children born to same parents are treated differently by the fathers. Some fathers make prompt payment of fees for some children and refuse to pay for others. Just because they believe such children are not their biological children. Sometimes men don’t even care about such pregnancies when they get the hint that their wives were impregnated by different men. Those children are known as* ***adinamo*** *or somebody born for him in Awutu dialect.”*

In some home deliveries, husbands were not allowed to assist because if a woman had ever had an extra marital affair, she may mention that in the event of a prolonged labour, out of fear based on beliefs. This could result in divorce. The belief in relation to confessing extra marital affairs was that if the woman failed to confess, she may suffer complications associated with prolonged labour or die in labour.

#### Beliefs in supernatural power

The belief in supernatural powers was also identified to be associated with male involvement. At least ten individual interviewees and six participants in FGDs explained that men who usually helped their wives to perform “women’s tasks” were regarded as having been machinated by their wives through supernatural means such as witchcraft or traditional medicine. It was believed that their wives might have prepared and administered “gbontor” or “abedianko” (love potion). In the FGD, a participant (35 years old and secondary school graduate driver) said:

“*Those of us who think we are helping to save the lives of our wives and children by performing household chores and accompanying them to clinics during pregnancy are given names that make us uncomfortable. Some will call you* ***yanoa ama wo*** *(they have cooked love potion for you). Even sometimes during meetings in the community or family meetings our contributions are considered as coming from our wives and we are not informed about important issues in the community. We have no respect and that is why some men feel like helping but cannot help*”*.*

### Community and organisational factors

The CHOs, CHVs and community leaders were interviewed on the strategies adopted to encourage men by removing the prejudices around male involvement in maternal healthcare. Even though visiting pregnant women has been part of the CHPS programme strategies, amongst the CHVs, only females were directly involved in paying visits to women.

“*The men in this CHPS zone only help to build and maintain the health facility through communal labour. The few who are CHVs are not fully involved in maternal health care. They leave that for the female CHVs whilst they concentrate on family planning. Perhaps, men find it very discomforting continuously getting closer to wives of others. We try to encourage them but it is difficult.*” *(CHO).*

Unlike the old system of healthcare, the CHPS system is focused on effecting changes in the rural healthcare system through active home visits by the CHOs and CHVs [21]. The study showed that the home visits aspect of the programme was not effective in the communities. It was only a few traditional birth attendants (TBAs) who paid frequent visits. According to some of the participants, the focus of the visits was on interacting with their wives who were either pregnant or breastfeeding newborns. Those who visited only sought permission from the men, as custom demands this, if they are to proceed with interacting with their wives.

It was reported by both the men and volunteers and confirmed by the CHOs that the workload for the CHOs was too high and they could not run the programme the desired way. In an IDI, a CHO shared this:

“*You know the CHVs are not paid and they have to work to survive. So we do not get their full attention as they are engaged in economic activities for livelihood. Some husbands do not cooperate and this discourages other men who are volunteers to put in their best. I must admit that the TBAs are doing great job in helping us. They help to identify problems for quicker intervention.*” *(CHO).*

The community volunteers who were mostly farmers also had to go to their farms to work for their livelihood. Almost all CHVs interviewed expressed the view that they were married men with children and accountable for the feeding and general welfare of their families. They therefore needed to work to be able to fulfil these responsibilities. One of them noted:

“*Sometimes the CHOs need our help when we are not around. We have to work as men to feed our wives, children and settle school fees and other bills. It is not our intention to leave CHPS programme activities to engage in other activities but a matter of survival. It is important to help save women's lives but we can't do this when we are hungry or unable to feed our families.*” *(CHV; 35 years old; farmer).*

The implementation of the maternal healthcare programme at the community level did not actively involve men. This was evident in all the five CHPS compounds. The occasional meetings to talk with men, without involving them directly, may not yield the desired results, especially at the household level where women need the most help. Thus, male support is necessary for women to progress through pregnancy and childbirth and to provide couples with the best opportunity for having healthy mothers and children [13].

The vision of CHPS is to accelerate progress towards Millennium Development Goals 4 and 5 on maternal health and child health [2]. The home visits by the CHOs encourage women and their families to develop interest in the use of modern health facilities and to continue using them. It also allows the CHOs to take time to explain certain important health issues with women and spouses in order to promote women’s health. Therefore, if men can be actively involved in maternal healthcare activities at the community level, especially during social gatherings and community health talks, they will play an active role at the household level as well. This is because they will be part of the decision-making process and will work closely with CHVs, CHOs and their wives to achieve the programme’s objectives. This way, they will serve as agents of change for themselves and for other men in the society [22].

In order to ensure active participation of the community members, and to establish leadership in health promotion, the planning aspect which used to be carried out at the district level is decentralised into the various CHPS zones [23]. This system therefore, calls for frequent interaction with the community members and since men or husbands are mostly the leaders in communities and families, their active involvement is essential.

### Socio-demographic factors

In both the IDIs and FGDs, most of the men below 40 years of age expressed the concern that they could not act contrary to societal norms by performing feminine roles. However, they indicated that their contributions were needed to save the lives of their wives and babies during the pregnancy-postpartum period. The data indicates that some of these younger husbands showed commitment to helping their wives in performing “women’s duties”, including accompanying them to the health centres for treatment. Some of the men in new marriages said, because they had no experience in pregnancy and childbirth, they followed the tradition by making their wives stay with their mothers to ensure they received the help necessary to avoid problems. Younger men in matriarchal societies, who were married to economically empowered women, were more involved in feminine gender roles. Of those husbands aged 40 years and above, most could cook for their families only during the period within the event when their wives were weak or medically advised not to work. Even though a few of them admitted to having accompanied their spouses to the health centres for treatment or check-ups, they emphasised that that was the work of women and they could not continue to do that work. One of them said:

“ *... and I only accompanied her to the clinic because her mother was not around, and all experienced women in my village had left for their farms. So I had no option*”*. (trader; 40 years old; Muslim and MSLC holder).*

Most men with secondary education and above were much more committed to assisting wives in household chores and accompanying them to the health centres. This concurs with previous studies which show that men who have formal education are able to break the walls of social and cultural norms around gender roles and help their spouses [22, 24]. On the other hand, this may be partially attributable to the fact that these men were married to women who were engaged in trading and vocational jobs such as seamstresses and beauticians. This category of women were mostly junior and senior high school leavers who were relatively economically empowered. This suggests that, in marriages where the traditional gender roles involving males being the providers and females being the full time home makers are broken, so too is the adherence to men’s and women’s roles in maternal care.

Men who did not accompany their wives to the clinic or help in household chores explained that they had to go to their farms, since delaying doing so could result in poor yields. Due to seasonality of rains, when the need to assist their wives coincided with the farming season, some husbands found it very difficult to help their spouses. Nonetheless, some husbands mentioned that they had to demonstrate love, care and responsibility by seeking the welfare of their wives. In fact, most of the farmers in both the IDIs and FGDs expressed this concern. In the FGD a participant noted:

“*Even though difficult I am able to help my wife because the doctor asked me to be doing so. The only barrier here is how our society classify roles for men and women, and the fact that farming is a seasonal job. If you delay working on your farm, you may plant late and lose when the rains cease*”*.(farmer; 27 years old; junior high school graduate).*

Regardless of the seasonality of farming and culture-based gender roles, some husbands explained that spending just one day or part of the day helping their spouses would not make any farmer unable to realise high yields. These few men claimed that maternal health issues were to be treated with the seriousness they deserve. These were the views of a husband in IDI:

“*We farm to feed our wives and children. So what is the sense in making higher yields and losing your wife or child or both during pregnancy or childbirth because we did not care to help them? Yes, I know some people say pregnancy is not a sickness but we must admit also that it is associated with complications and death*” *(A farmer aged 50 who is a middle school leaver)*

Religious affiliation appears to have had some influence on the nature of male involvement, as illustrated by the views of a man who is a Christian:

“*I think pregnancy and childbirth are part of* ‘*for better for worse*’ *as indicated in the Bible. When a man and a woman get married they become one and so must suffer together. If you are ready to marry you must be ready to bear the consequences of marriage. Who should enjoy for whom to bear the pain?*” *(Teacher, 34 years; Christian and a diploma holder).*

Irrespective of societal norms and expectations, men with secondary school level education and above were able to overcome traditional gender norms, ignore gender norms and assume feminine roles towards improvement of maternal health. Men of Christian faith were more directly involved in the feminine roles than members of other religions. To them, it was their responsibility to assist their wives to perform any role, especially during pregnancy. This might explain the higher maternal clinical attendance amongst the Christians in this district [25].

The study observed that; some men preferred helping their spouses by using their own mothers and sisters rather than the wives’ relatives. However, the men with two or more wives used the other wives instead of relatives to take care of their pregnant wives or nursing mothers and their babies. Only after delivery could the women choose to stay with her own mothers, as is the usual practice**.** This practice was to enable the nursing mother and her baby to get adequate attention from the maternal grandmother and to ensure that the woman was fully healed before going back to her matrimonial home. This was a response in FGD which was supported by most men who were in polygamous marriages:

“*Why should I disturb myself when I know it is woman who can help woman. I married two. So if one is pregnant other woman will help. That is why we say a man should have at least two wives. But if a man is not able to marry two wives he can use his own female relatives rather than the wife's relative*” *(42-year-old man; Muslim; trader; senior high school graduate).*

The men assisted their wives to play the feminine gender roles by using their female relatives and other wives who could occupy the space to perform those tasks on their behalf. In both FGDs and IDIs, some participants stated that it was not appropriate to impregnate a woman and later send her to her mother to suffer. They further argued that in polygamous marriages there should be reciprocal assistance between/amongst wives of a husband so that whenever one of them is pregnant or sick, the other(s) can help. In justifying polygamy, they added that a man with one wife is like a bachelor and this is felt when the wife is pregnant, sick or when she travels.

The data showed that men who were in socially recognised relationships - that is those who followed customary, ordinance or Islamic marriage procedures were more helpful, concerned and showed more care in helping spouses to ensure good health. However, a similar study observed that formally married men and those in cohabitational relationship behaved similarly [13]. Irrespective of the cultural norms and other obstacles, the men in single marriages were more involved in helping their spouses at the household level, and in encouraging and accompanying them to clinics.

This study observed that the men were not necessarily indifferent towards maternal healthcare. Rather, they were directly involved in activities such as building CHPS compounds, supervising drug intake and bearing the cost of healthcare, as well as engaging female relatives or co-wives to render services such as escorting wives to health facilities and household chores which are regarded as women’s space in Ghana [7–9, 13, 20, 26, 27]. This finding concurred with a study in Ghana [13] which showed that men were mostly involved in supporting their spouses financially to seek ANC but, contrary to the annual health sector review report [5], that men were solely involved in communal labour in the CHPS zones. The internalisation and normalisation of traditional gender roles in the communities created obstacles to husbands, including the male CHVs who worked at the community level with the CHOs in the CHPS compounds, actively carrying out the traditionally tagged female roles.

## Conclusions

The study showed conflicting data on what constitutes male involvement, as the nature of involvement varied. Some husbands were actively involved in both traditional masculine and feminine roles, whilst others did not want to be directly involved in traditional feminine roles but made provision for female relatives and co-wives to perform such tasks. It is clear that the men were not necessarily indifferent towards maternal healthcare, but rather, they operationalised their care within the spaces allowed for them by the society. Men who were directly involved tended to be those who were young, educated, Christians and men who were in formal monogamous relationships. Those who were not directly involved but made arrangements for others to fulfil the roles were Muslims in polygamous marriages in patriarchal societies, men over 40 years old and those with little formal education.

The study highlighted that socio-cultural norms exerted a strong influence on the perceptions and behaviours of men. It needs to be highlighted that women themselves were perpetuating strong gender expectations which limited male involvement in certain domains. These domains of male involvement should be considered in health promotion planning and implementation.

Male involvement in maternal healthcare therefore needs to be understood in the broader frameworks of gender relations in matriarchal and patriarchal societies, taking into account religious differences as well as educational and generational demarcations. The questions that need to be raised are: what constitutes male involvement and whose definition of male involvement should be used when we consider the contribution of men in maternal healthcare? It is important to listen to the views of men so as to understand the nature of their involvement. Bearing in mind that men are very influential in positive health outcomes for pregnancy and childbirth, to acknowledge their contribution in the way they have been involved is a step in the right direction in programme planning and implementation. To impose a predetermined benchmark of male involvement is not only discounting their contributions, but may also further alienate them in maternal health care.

Lastly, the design and planning of male involvement programmes in maternal health care should also consider the types, age and the bond of intimacy of marriages. Needs assessment of couples, which can be conducted with improved communication between provider and service users, will lead to more meaningful healthcare intervention programmes or policies catering for the varied socio-cultural needs of the couples. This will serve as a first step towards facilitating the provision of effective counselling and supportive services instead of the “one-size fits all” programmes hitherto applied in health facilities.

## Abbreviations

ANC: Antenatal care
CHO: Community health officer
CHPS: Community-based health planning and services
CHV: Community health Volunteer
CHW: Community health worker
CRS: Catholic relief services
FGD: Focus group discussion
ICRW: International centre for research on women
IDI: Individual interview
MDG: Millennium development goal
PNC: Postnatal care
UNFPA: United Nations population fund
USAID: United States agency for international development
WHO: World health organisation
